# Comparative Analysis of the Differences in Dentofacial Morphology According to the Tongue and Lip Pressure

**DOI:** 10.3390/diagnostics11030503

**Published:** 2021-03-12

**Authors:** Yoo-Sun Lee, Jiho Ryu, Seung-Hak Baek, Won Hee Lim, Il-Hyung Yang, Tae-Woo Kim, Seok-Ki Jung

**Affiliations:** 1 Department of Orthodontics, School of Dentistry, Dental Research Institute, Seoul National University, Seoul 03080, Korea; yousunzzang@snu.ac.kr (Y.-S.L.); jiho@snu.ac.kr (J.R.); drwhite@snu.ac.kr (S.-H.B.); whlim@snu.ac.kr (W.H.L.); drortho@snu.ac.kr (I.-H.Y.); 2 Department of Orthodontics, Korea University Ansan Hospital, Ansan, Gyeonggi 15355, Korea

**Keywords:** tongue pressure, lip pressure, dentofacial morphology, iowa oral performance instrument

## Abstract

The aim of this study was to evaluate the effects of the tongue and lip pressure on dentofacial morphology. The subjects comprised 194 patients with malocclusion. Anterior and posterior tongue elevation and lip pressures were evaluated using the Iowa Oral Performance Instrument (IOPI) device. The lateral cephalograms of each subject were traced and digitized to perform the analysis. Statistical analysis was used to investigate the relationship between perioral muscle force and the cephalometric variables. Anterior and posterior tongue pressure was both higher in males than in females. No sex difference in lip pressure was observed. The group with a low posterior tongue pressure showed a short ramus height, short posterior facial height, and clockwise-rotated mandible. On the other hand, lip pressure had a significant influence on maxillary incisor angulation. Skeletal pattern was not found to be significantly related with lip pressure. The anterior tongue pressure appeared as a mixed pattern of the two results. Tongue pressure was related to skeletal measurements, such as short posterior facial height, and lip pressure was related to the angulation of the anterior teeth. This study suggests that there may be differences in dentofacial morphology according to the differences in perioral muscle force.

## 1. Introduction

The balance between the tongue and perioral muscle is known to affect the position of the teeth, and it plays a significant role in the formation and maintenance of the dental arch form [1,2,3,4]. Due to the fact that the teeth are positioned between the lips, cheeks, and tongue, the force from this muscle is the major factor that determines the tooth position. It is important to evaluate the dynamics of the perioral muscle at rest and during function, as the function of the perioral muscle is closely related to the onset of malocclusion [5,6,7]. In addition, the contribution of the strength of the lips, cheeks and tongue is important to orthodontists for proper treatment planning and achieving posttreatment stability [8].

The tongue is a powerful muscular organ that exerts pressure at frequent intervals during the day and night, performing various functions, such as mastication, swallowing, and phonation. Several studies have reported the effect of tongue size on the dental arch [4,9,10]. Proffit stated that the resting position of the tongue is among the key factors in the maintenance of the dental equilibrium [11]. It has been shown that the tongue posture is lower in skeletal class III patients than in class I patients [3].

However, previous studies regarding the effect of tongue pressure on the dentofacial morphology have shown different results. Kurabeishi et al. showed that maximum tongue pressure is significantly lower in the skeletal class II group than in the skeletal class III group [12]. On the other hand, several studies have suggested that tongue pressure is not related to the craniofacial morphology [13,14]. The limitations of these studies were that they measured only the anterior pressure of the tongue tip, and only the anteroposterior skeletal difference was evaluated. Despite the many previous studies on tongue pressure, the relationship between dentofacial morphology and tongue pressure remains uncertain [13,15,16].

Some studies have been conducted to understand the effect of lip closure force on the onset of malocclusion [14,17]. Jung et al. showed that the upper incisor inclination is related to the lip-closing force in males with normal molar occlusion [18]. Other studies have reported that the lip-closing force produced by subjects of angle class II division 1 are weaker than those of angle class I relationships [2].

However, in previous studies, a self-made measuring device or a modified sensor has been used to measure the pressure at a specific intraoral location. Due the fact that the measurement methods are not standardized, results cannot be compared.

Therefore, the aim of this study was to evaluate the effects of the tongue and lip pressure on dentofacial characteristics using a standardized measuring device. Since the contribution of tongue and lip pressure is an important part in the treatment planning, the significance of this study can be found.

## 2. Materials and Methods

### 2.1. Subjects

The sample consisted of 194 patients who visited the department of orthodontics at Seoul National University Dental Hospital. At the initial orthodontic consultation, an informed consent was obtained from the patients. Patients with maxillofacial deformities and neurological disorders were excluded. The sample included 104 men and 90 women (mean age: 23.65; SD: 7.3).

### 2.2. Measurement Methods of the Lateral Cephalometric Radiographs

Cephalometric radiographs were taken with the CX-90SP cephalostat (Asahi Co, Kawasaki, Japan), 72 to 74 k (peak), 20 mA/sec, at the Department of Oral Maxillofacial Radiology in Seoul National University Dental Hospital. Each radiograph was taken in the natural head position. The natural head position is a reproducible position of the head in an upright posture, and the eyes look at the point of the eye level position. In addition, all patients were instructed to hold their breath and not swallow while the radiographs were being taken.

**Figure 1:**
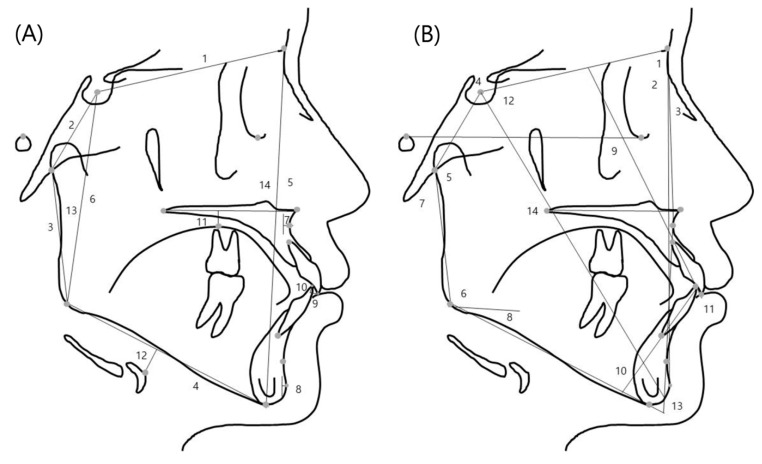
(**A**) Linear variables used in this study: 1, anterior cranial base (ACB); 2, posterior cranial base (PCB); 3, ramus height (RH); 4, mandibular body length (MBL); 5, anterior facial height (AFH); 6, posterior facial height (PFH); 7, point A to N perpendicular (A to N-perp); 8, pogonion to N perpendicular (Pog to N-perp); 9, overjet (OJ); 10, overbite (OB); 11, dorsum of the tongue to palatal plane (Tp to PP); 12, hyoidale to mandibular plane (H to MP); 13, body to anterior cranial base ratio (body to ACB); 14, facial height ratio (FHR). (**B**) angular variables used in this study: 1, SNA angle; 2, SNB angle; 3, ANB angle; 4, saddle angle; 5, articular angle; 6, gonial angle; 7, Björk sum; 8, Frankfort horizontal plane to mandibular plane angle (FMA); 9, upper incisor to FH angle (U1 to FH); 10, incisor mandibular plane angle (IMPA); 11, inter-incisor angle (IIA); 12, Y-axis to SN angle; 13, AB plane to mandibular plane angle (AB to MP); 14, palatal plane to Frankfort horizontal plane angle (PP to FH).

A single investigator performed all the tracings. The reference points were digitized with V-ceph (ver 5.3, Osstem Inc., Seoul, Korea). Twenty landmarks and 28 measurements were used in this study (Figure 1).

### 2.3. Tongue and Lip Pressure Measurements

Oral muscle strength measurements were performed using the Iowa Oral Performance Instrument (IOPI; Medical LLC, Carnation, WA, USA). The Iowa Oral Performance Instrument (IOPI) objectively measures the tongue and lip strength [19]. The IOPI measures the strength of the tongue by measuring the maximum pressure that an individual can produce in a standard-sized air-filled bulb by pressing the bulb against the roof of the mouth with the tongue. The peak pressure achieved is displayed on a liquid-crystal display (LCD). The units displayed are kilopascals (kPa), based on the internationally recognized unit of pressure, the Pascal (Pa). Currently, it is among the most commonly used measurement techniques available, and it has been validated in many previous studies [20,21].

**Figure 2:**
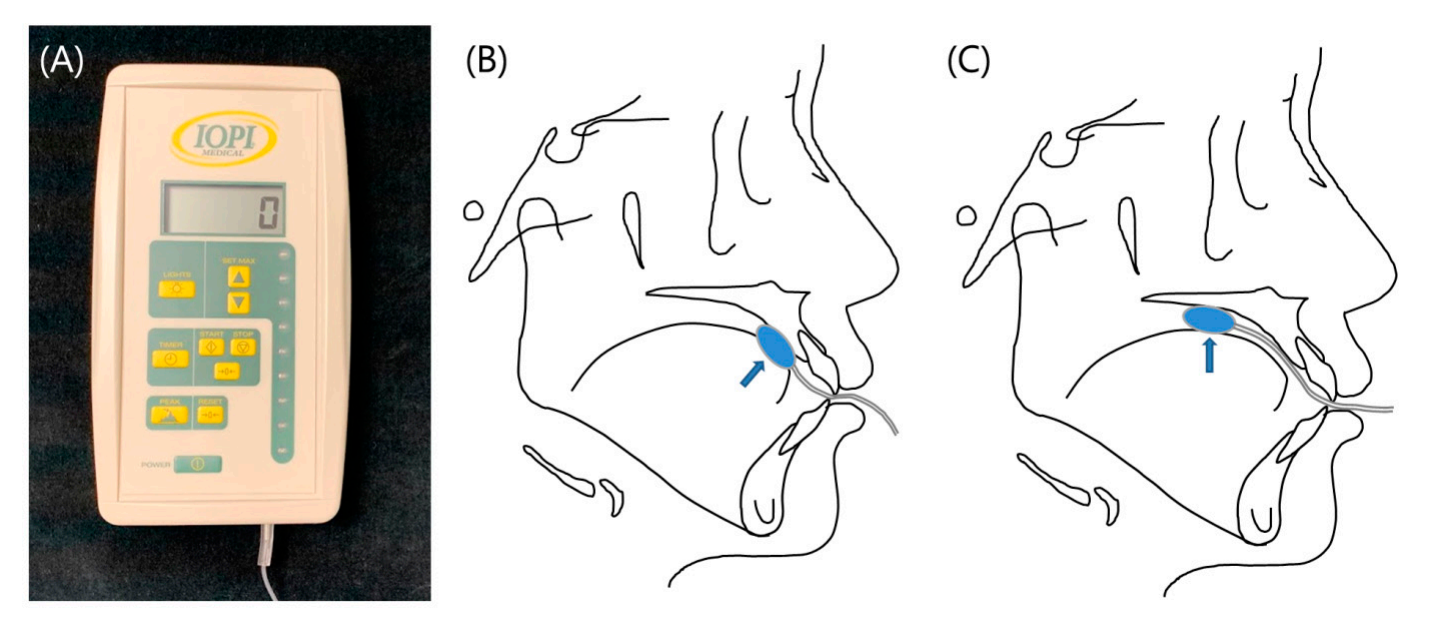
(**A**) Iowa Oral Performance Instrument (IOPI) device. (**B**) Position of the bulb (TAP, anterior tongue pressure). (**C**) Position of the bulb (TPP, posterior tongue pressure).

**Figure 3:**
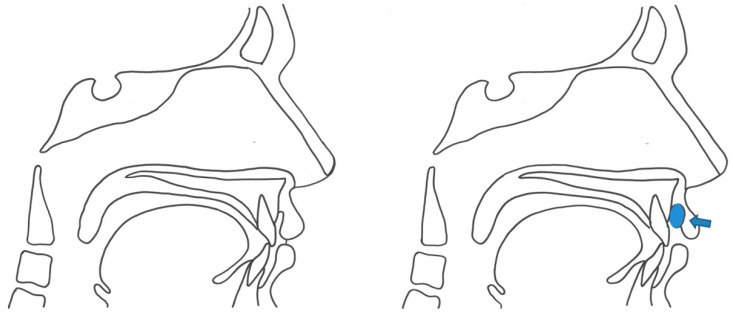
Position of the bulb (LP, lip pressure).

The bulb was positioned longitudinally on the hard palate just posterior to the alveolar ridge to measure the anterior tongue pressure (TAP). Posterior tongue pressure (TPP) was measured with the bulb positioned more posteriorly, with the distal end of the bulb at the posterior border of the hard palate (Figure 2). After that, the bulb was positioned horizontally along the upper vestibule, which is between the upper lip and the gums and teeth to measure the lip pressure (LP) (Figure 3).

Patients were instructed to press the bulb as strongly as possible without biting the teeth and not to change the position of the bulb for each measurement. Three measurements were conducted for each patient at intervals of 30 s, and the average value was used.

### 2.4. Statistical Analysis

To investigate whether the perioral muscle forces (TAP, TPP, and LP) were different according to sex, a t-test was performed. Pearson’s correlation test was performed to analyse the correlation of cephalometric variables with perioral muscle forces. For each perioral muscle force (TAP, TPP, and LP), the upper/lower groups were divided based on the average value: 42 kPa for anterior tongue pressure, 40 kPa for posterior tongue pressure, and 24 kPa for lip pressure (Table 1). Group comparisons between the high force group and low force group were performed using a *t*-test. Multiple linear regression analysis was used to establish the relationship between tongue and lip force and the cephalometric variables. The regression analysis was conducted three times by setting TAP, TPP, and LP as a response variable, respectively. Explanatory variables were age and 28 cephalometric measurements. The stepwise variable selection technique was used to reduce the number of explanatory variables and to select the significant ones. Statistical analyses were performed using SPSS software (ver 25.0; IBM Corp., Armonk, NY, USA). Statistical significance was accepted at *p* < 0.05.

## 3. Results

**Figure 4:**
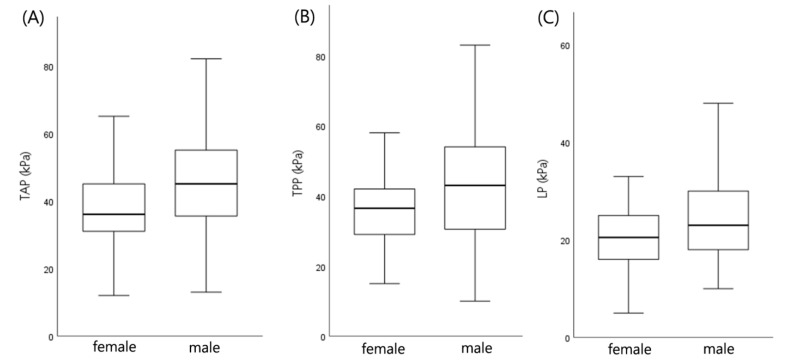
Sex-based pressure differences; (**A**) TAP, anterior tongue pressure. (**B**) TPP, posterior tongue pressure. (**C**) LP, lip pressure.

There was a statistically significant difference in tongue pressure between males and females (*p* < 0.05). Anterior and posterior tongue pressures were both higher in males (Figure 4). Meanwhile, no sex difference in lip pressure was observed.

In the Pearson’s correlation test, posterior cranial base (PCB) and posterior facial height (PFH) showed the highest positive correlation, and overjet (OJ) showed the highest negative correlation with anterior tongue pressure. Additionally, PCB and posterior facial height (PFH) showed the highest positive correlation with posterior tongue pressure. The angulation of the upper incisor to FH plane (U1 to FH) showed a negative correlation with lip pressure (Table 2).

Comparisons of the cephalometric measurements between the upper/lower groups are given in Table 3, Table 4 and Table 5. Variables that were different among groups were articular angle, PCB, OJ, and H to MP for anterior tongue pressure, and Björk sum, PCB, PFH, FHR, and ramus height for posterior tongue pressure. In the group with a low anterior tongue pressure, the articular angle and OJ were large, and the PCB and H to MP were short. In the group with a low posterior tongue pressure, the length of PCB, PFH, and ramus height were short, and the Björk sum and FHR were small.

In the case of lip pressure, OJ, IIA, and PCB showed significant differences between groups. The group with a low lip pressure tended to have a large OJ and small IIA and PCB. This shows that the pressure on the upper lip mainly affects the angle of the anterior teeth, not the skeletal-related measurements. However, the SDs for the OJ were high, showing a statistically significant difference, but showed high variability within the groups.

The results of the multiple linear regression analysis are summarised in Table 6. As shown in the table, anterior tongue pressure was significantly different according to the PCB and OJ. The regression coefficients indicated that an increase in anterior tongue pressure would be observed, as the PCB increased and OJ decreased. In contrast, a significant difference in posterior tongue pressure was observed according to PCB, Tp to PP, OJ, and age. The greater the distance from the palatal plane to the dorsum of tongue, the lower the posterior tongue pressure value. Regarding lip pressure, a decreasing tendency of the lip pressure was observed as the angle U1 to FH and FMA were increased.

**Figure 5:**
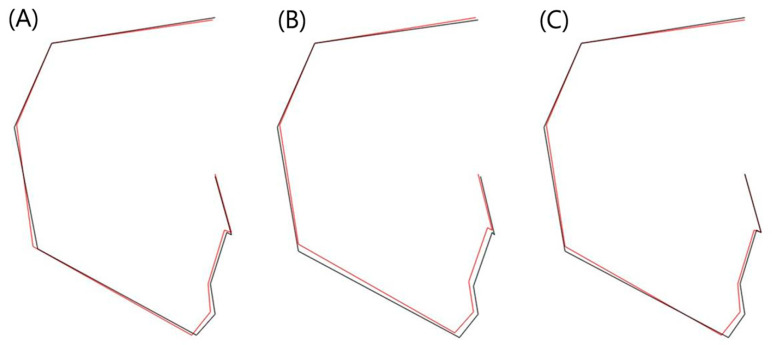
Comparison of skeletal profilograms between the high group (black line) and the low group (red line); (**A**) TAP, anterior tongue pressure. (**B**) TPP, posterior tongue pressure. (**C**) LP, lip pressure.

Comparing the skeletal profilograms, it can be seen that the low TPP group showed characteristics of short ramus height and short PFH, and the low LP group showed a large OJ and small IIA, which indicates the proclination of the central incisors (Figure 5). In addition, the low TAP group exhibited characteristics of both groups (low TPP and low LP group).

## 4. Discussion

Results obtained from previous studies regarding tongue pressure cannot be compared to one other because the measurement locations are different, and the measurement methods were not standardized. In this study, we used the Iowa Oral Performance Instrument (IOPI), an appropriate evaluation tool, to objectively measure the tongue strength [19]. In order to reduce the error when the force was not accurately applied during the first measurement, three measurements were performed, and the average value was used.

Anterior and posterior tongue pressures were both higher in males. As reported in previous studies, it is thought to be the result of basic muscle mass differences between men and women [21,22]. The study investigating the orofacial strength (tongue, lip, and buccinator muscle) of healthy adults showed that men had a greater strength than women in all measurements [22].

From the results of the correlation analysis of the perioral muscle force and cephalometric variables, anterior and posterior tongue pressure showed a positive association with PCB, RH, PFH, FHR, and SNB, and a negative association with Björk sum and Y-axis to SN. According to these findings, patients with a vertical skeletal pattern who have a short ramus height and a clockwise-rotated mandible tend to have weak tongue strength. Variables related to the maxillary incisor angulation (U1 to FH and IIA) showed significant correlations with lip pressure. Dentofacial morphology according to the differences in the anterior tongue pressure showed a mixed tendency of dentofacial morphology according to the posterior tongue pressure and lip pressure. Anatomically, the upper and lower incisors are located between the tongue and the lips; therefore, if the anterior tongue pressure is strong and the lip pressure is weak, the maxillary anterior teeth are proclined, and the OJ becomes larger.

Through a comparative analysis between groups, skeletal pattern and skeletal size were found to be related to the tongue pressure. In particular, short posterior facial height and clockwise-rotated mandible were seen in the group with a low posterior tongue pressure. This is probably due to similar reasons, i.e., masticatory muscle strength tends to be low in the group with a skeletal open bite [23]. This shows that the pressure on the lips correlates with the angle of the anterior teeth.

In the multiple regression analysis between the perioral muscle force and the cephalometric variable, the statistically significant independent variables were PCB, Tp to PP, age, OJ, U1 to FH, and FMA. Anterior and posterior tongue pressure exhibited positive associations with PCB and negative associations with OJ. Lip pressure was found to be negatively associated with U1 to FH. Higher lip pressure results in the inclination of the upper incisors lingually. Our finding was consistent with that of a previous study in which subjects with Angle Class II division 1 relationships showed a lower lip-closing force than those with Angle Class I relationships [2].

The limitation of this study was that only upper lip pressure was evaluated, and the role of the lower lip pressure remained unknown. Another limitation was that only two functional parameters of the tongue were examined (TAP and TPP), whereas there was no assessment of the same parameters at rest. This could also be a suggestion for future studies. Tongue posture and function have been associated with the aetiology of malocclusions and posttreatment stability. Smithpeter demonstrated that combined myofunctional therapy has shown better results in long-term maintenance in anterior open bite closure than orthodontic treatment alone [24]. Meazzini et al. reported that the presence or absence of a tongue with glossectomy did not affect mandibular growth in patients with Beckwith-Wiedermann syndrome [25]. Once the effects of orofacial myofunctional therapy (OMT) on the tongue strength have been identified, they will be used as scientific evidence to support the importance of OMT. The treatment of malocclusion could probably benefit from the establishment of harmonious relationships between the perioral muscles. It is also necessary to study how perioral muscles affect the growth pattern in the future.

## 5. Conclusions

Anterior and posterior tongue pressures were higher in males than in females. The group with a low posterior tongue pressure had a short posterior facial height, short ramus height, and clockwise-rotated mandible. Lip pressure affects the angle of the anterior teeth, not the skeletal-related measurements. Higher lip pressure results in the inclination of the upper incisors lingually. Anterior tongue pressure showed a mixed tendency of posterior tongue pressure and lip pressure. The group with a low anterior tongue pressure had a large OJ and short posterior cranial base length.

## Figures and Tables

**Table 1:** Sex and age distribution of patients with each group.

		TAP (kPa)		TPP (kPa)		LP (kPa)	
		Low (<42)	High (≥42)	Low (<40)	High (≥40)	Low (<24)	High (≥24)
Sex	Male	45	59	46	58	57	47
	Female	55	35	57	33	62	28
Age (y)	Mean	23.2	24.2	22.6	24.8	23.3	24.2
	SD	6.9	7.7	6.6	7.8	7.2	7.4

TAP, anterior tongue pressure; TPP, posterior tongue pressure; LP, Lip pressure.

**Table 2:** Correlation coefficients between cephalometric variables and perioral muscle force (TAP, TPP, LP) †.

	TAP		TPP		LP	
Cephalometric Variable	Correlation	*p*-Value	Correlation	*p*-Value	Correlation	*p*-Value
ACB	0.151	0.035 *	NS	NS	NS	NS
PCB	0.277	<0.001 ***	0.229	0.001 **	NS	NS
RH	0.205	0.004 **	0.147	0.041 *	NS	NS
PFH	0.251	<0.001 ***	0.201	0.005 **	NS	NS
Pog to N-perp	0.147	0.04 *	NS	NS	NS	NS
OJ	−0.202	0.005 **	NS	NS	NS	NS
FHR	0.183	0.011 *	0.19	0.008 **	NS	NS
SNA	NS	NS	0.149	0.038 *	NS	NS
SNB	0.161	0.025 *	0.16	0.026 *	NS	NS
ANB	−0.151	0.035 *	NS	NS	NS	NS
Articular angle	−0.151	0.036 *	NS	NS	NS	NS
Björk sum	−0.153	0.033 *	−0.174	0.015 *	NS	NS
U1 to FH	NS	NS	NS	NS	−0.231	0.001 **
IIA	NS	NS	NS	NS	0.211	0.003 **
Y axis to SN	−0.146	0.042 *	−0.146	0.042 *	NS	NS

† Only shows the variables for which a significant correlation was found.NS, not significant. TAP, anterior tongue pressure. TPP, posterior tongue pressure; LP, Lip pressure. * *p* < 0.05, ** *p* < 0.01, *** *p* < 0.001.

**Table 3:** Comparisons of cephalometric variables between TAP_low and TAP_high groups (n = 194). *

Cephalometric Variables	TAP_Low	TAP_High	Significance	Comparisons
Articular angle (°)	148.2 ± 8.0	146.0 ± 7.4	0.022 *	TAP_low > TAP_high
PCB (mm)	38.6 ± 3.6	39.7 ± 4.2	0.027 *	TAP_low < TAP_high
OJ (mm)	2.5 ± 4.0	1.3 ± 3.8	0.016 *	TAP_low > TAP_high
H to MP (mm)	13.0 ± 5.6	14.7 ± 5.9	0.019 *	TAP_low < TAP_high

* Only shows the variables for which a statistically significant difference was found (*p* < 0.05). TAP_low, low anterior tongue pressure group; TAP_high, high anterior tongue pressure group. * *p* < 0.05.

**Table 4:** Comparisons of cephalometric variables between TPP_low and TPP_high groups (n = 194). *

Cephalometric Variables	TPP_Low	TPP_High	Significance	Comparisons
Björk sum (°)	398.2 ± 7.4	396.4 ± 7.2	0.045 *	TPP_low > TPP_high
PCB (mm)	38.5 ± 3.6	39.8 ± 4.3	0.011 *	TPP_low < TPP_high
PFH (mm)	87.2 ± 8.8	90.2 ± 9.0	0.010 *	TPP_low < TPP_high
FHR	63.9 ± 5.6	65.6 ± 5.9	0.021 *	TPP_low < TPP_high
RH (mm)	52.5 ± 7.1	54.5 ± 7.7	0.031 *	TPP_low < TPP_high

* Only shows the variables for which a statistically significant difference was found (*p* < 0.05). TPP_low, low posterior tongue pressure group, TPP_high, high posterior tongue pressure group. * *p* < 0.05.

**Table 5:** Comparisons of cephalometric variables between LP_low and LP_high groups (n = 194). *

Cephalometric Variables	LP_Low	LP_High	Significance	Comparisons
IIA (°)	125.3 ± 11.9	128.8 ± 13.7	0.034 *	LP_low < LP_high
PCB (mm)	38.7 ± 3.8	39.7 ± 4.1	0.045 *	LP_low < LP_high
OJ (mm)	2.4 ± 3.9	1.1 ± 3.9	0.013 *	LP_low > LP_high

* Only shows the variables for which a statistically significant difference was found (*p* < 0.05). LP_low, low lip pressure group. LP_high, high lip pressure group. * *p* < 0.05.

**Table 6:** Stepwise multiple regression analysis for the perioral muscle force.

Dependent Variable	Statistically Significant Independent Variables	B	SE B	*p*-Value	Adjusted R-Quared	F-Statistic
TAP	PCB (mm)	0.86	0.24	<0.001 ***	0.091	10.62
	OJ (mm)	−0.55	0.25	0.027 *		
TPP	PCB (mm)	0.92	0.25	<0.001 ***	0.099	6.28
	Tp to PP (mm)	−0.56	0.21	0.01 *		
	OJ (mm)	−0.53	0.26	0.04 *		
	age	0.29	0.13	0.03 *		
LP	U1 to FH (°)	−0.48	0.11	<0.001 ***	0.095	11.14
	FMA (°)	−0.45	0.14	0.001 **		

TAP, anterior tongue pressure; TPP, posterior tongue pressure; LP, Lip pressure; B, unstandardized regression coefficient; SE B, standard error of B. * *p* < 0.05, ** *p* < 0.01, *** *p* < 0.001.

## Data Availability

The data underlying this article will be shared on reasonable request from the corresponding author.
